# Was Pauling Mistaken about Metals?

**DOI:** 10.3390/molecules26071930

**Published:** 2021-03-30

**Authors:** Andreas Savin

**Affiliations:** Laboratoire de Chimie Théorique, CNRS and Sorbonne University, 4 Place Jussieu, CEDEX 05, 75252 Paris, France; andreas.savin@lct.jussieu.fr

**Keywords:** Kronig–Penney model, maximum probability domains, metallic bond, resonant structures

## Abstract

Pauling described metallic bonds using resonance. The maximum probability domains in the Kronig–Penney model can show a picture of it. When the walls are opaque (and the band gap is large) the maximum probability domain for an electron pair essentially corresponds to the region between the walls: the electron pairs are localized within two consecutive walls. However, when the walls become transparent (and the band gaps closes), the maximum probability domain can be moved through the system without a significant loss in probability.

## 1. Introduction

Years ago, I met a famous physicist (whose name should not be divulged here). He said that Pauling had many good ideas, but that he had been wrong about one thing: metals. Of course, there are many aspects related to metals, some of which go beyond their electronic structure; these are of interest in the present paper. So, how did Pauling see metals? In his *Nature of the chemical bond and the structure of molecules and crystals* [1], he describes them as he describes molecules: in terms of resonant structures. For lithium metal, he schematizes it as:

$$\begin{array}{ccccccccccccc} \mathrm{Li} & - & \mathrm{Li} & & \mathrm{Li} & & \mathrm{Li} & & \mathrm{Li} & - & {\mathrm{Li}}^{-}\\ & & & ↔ & | & & | & ↔ & & & | & ↔ & \cdots \\ \mathrm{Li} & - & \mathrm{Li} & & \mathrm{Li} & & \mathrm{Li} & & {\mathrm{Li}}^{+} & - & \mathrm{Li} \end{array}$$

and notes the analogy to benzene.

Instead of performing valence bond calculations for metals, this paper searches for electron pairs in the very simple model of Kronig and Penney [2]. It is the textbook model for explaining the band structure. This model has important limitations, including that it is only one-dimensional and deals with independent electrons. However, by modifying a single parameter, we can switch between insulator and metal.

We search for a domains, maximizing the probability to find a pair of electrons in themWe find that in the insulator regime, these domains are well-defined in space, while in the metallic regime a translation produces an equivalent domain.

## 2. Theory

### 2.1. The Model of Kronig and Penney

**Figure 1:**
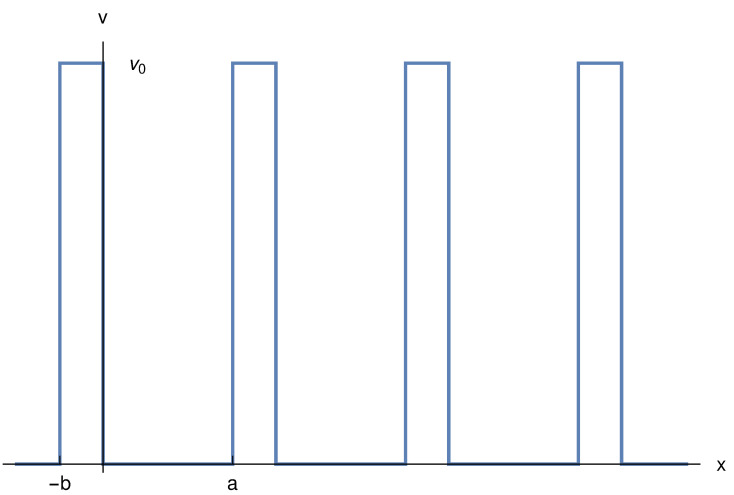
The potential in the model of Kronig and Penney [2]. In this paper, we consider the case $b\to 0,v_{0}\to \infty$, such that $b\;v_{0}=\Omega$.

In the model of Kronig and Penney [2], one deals with non-interacting electrons moving in the periodic one-dimensional potential, as shown in Figure 1:

$$v=\left\{\begin{array}{cc} 0 & \mathrm{if}x\in (0,a)\\ v_{0} & \mathrm{if}x\in (a,a+b)\\ \cdots \end{array}\right.$$

It has walls of thickness *b* and height $v_{0}$. In order to reduce the number of parameters, we consider, as Kronig and Pennig also did, the limit obtained when b→0, $v_{0}\to \infty$, such that $b\;v_{0}=\Omega$. We also choose as the distance between two walls a=1. This brings no loss of generality, as it only corresponds to a change in units. The total length (and, with our choice of a,b the total number of units) is *L*. Ω can be seen as the opacity of the wall. When Ω→∞, the particles are confined between the walls. When Ω→0, the walls disappear and the particles are free to move over the whole domain.

The energy levels, ε, are quantized, and, as *L* increases, bands are formed. A continuum of levels appears between ε=0 and $\varepsilon =\pi^{2}/2$. For Ω=0, there is no gap between this band and the next one. When Ω≠0, a gap opens up. It increases with Ω, up to $3\pi^{2}/2$, for Ω=∞. In the following, we will consider the levels filled up to $\varepsilon =\pi^{2}/2$.

### 2.2. Maximizing the Probability

In order to connect with bonds, we determine the interval $D=(x_{\min},x_{\max})$ for which the probability of finding a pair of electrons is maximal [3,4]. As we are dealing with independent electrons, we may as well search for the interval D, such that the probability to find a single electron in it, *P*, is maximal. We thus occupy each of the energy levels up to $\pi^{2}/2$ with a single electron of a given spin. Occupying it with other spin yields the same probability, and because the electrons are independent, the probability of finding a pair is the square of that of finding a single electron. For these N=L electrons, the probability to find one, and only one, electron in D is given by:

(1) $$P\left(D\right)=N\int_{D}dx_{1}\int_{\bar{D}}dx_{2}\ldots dx_{N}{\left|\Psi \left(x_{1},\cdots ,x_{N}\right)\right|}^{2}$$

where $\bar{D}$ is the domain that excludes D. The prefactor *N* arises because the electrons are not distinguishable. A numerically efficient way to compute this was given by E. Cancès [5]. The maximal value of *P* is $P_{\max}$:

(2) $$P_{\max}=\max_{D}P\left(D\right)$$

The corresponding domain (the maximum probability domain) is $D_{\max}$:

(3) $$D_{\max}=\arg\max_{D}P\left(D\right)$$

All computations were conducted with Mathematica [6].

## 3. Results

### 3.1. Choosing the Size of the System

**Figure 2:**
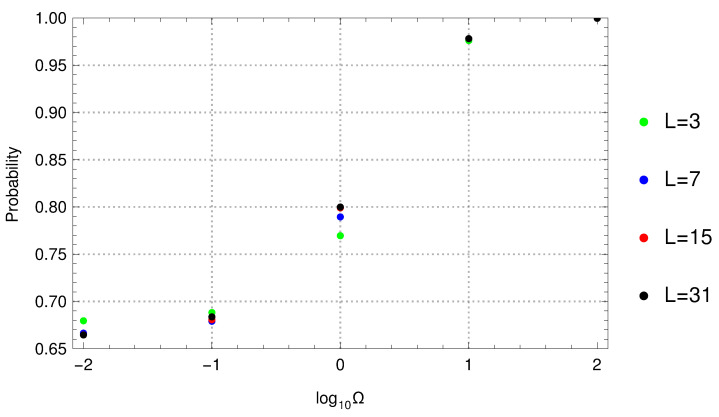
The maximal probability to find an electron in an interval, $P_{\max}$, Equation (2), for different values of opacity, Ω, and for different numbers of units, *L*, in the model of Kronig and Penney [2].

In order to study crystals, we should let the number of cells, *L*, become very large. However, in computing the probability, $P_{\max}$, Equation (2), becomes more time-consuming as *L* increases. Fortunately, the convergence with *L* is fast, as can be seen in Figure 2 where $P_{\max}$ is shown for different values of *L* and Ω. Note that already L=7 gives a reasonable estimate for $P_{\max}$; this is the value for *L* chosen below.

### 3.2. How the Probability Depends on the Opacity

Figure 2 shows how *P* changes with the opacity, Ω. When Ω=∞, the electron cannot escape the unit cell, and no other electron can get in: P=1. As Ω decreases, the electron can tunnel through the walls. However, even for Ω=0, there is a domain D that maximizes the probability: the Pauli principle is still active, and it keeps the electrons apart.

### 3.3. The Maximum Probability Domain

**Figure 3:**
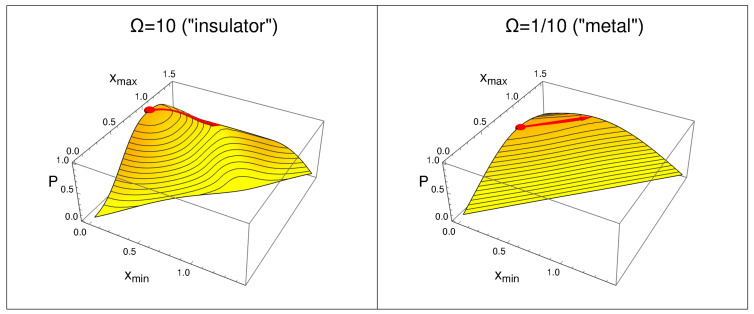
Probability to find an electron between $x_{\min}$ and $x_{\max}$, P(D), Equation (1), for large opacity of the wall (Ω=10, left, “insulator”), and small opacity (Ω=1/10, right, “metal”). The small red sphere indicates $P_{\max}$, Equation (2). The arrow follows a translation of the maximum probability domain, $D_{\max}$, Equation (3), strongly changing the probability *P* for large Ω, weakly changing it for small Ω.

Let us now turn our attention to the domain maximizing *P*, $D_{\max}$, Equation (3). Figure 3 shows the value of *P* as a function of $x_{\min}$ and $x_{\max}$. Independently of the opacity, Ω, one finds that it is defined by an interval between $x_{\min}\approx 0$, and $x_{\max}\approx 1$. As in Figure 2, we see that the maximal value of *P* increases with Ω. Note that another feature is present, too. Meanwhile, for large values of Ω the maximum of $P_{\max}$ is well characterized; for small values of Ω, a translation of the domain $D_{\max}$ by some δ — i.e., $x_{\min}\to x_{\min}+\delta$ and $x_{\max}\to x_{\max}+\delta$ — has almost no effect. Of course, when the wall disappears, translational invariance requires that there strictly is no effect when translating by some δ.

## 4. Conclusions

### 4.1. Summary

We are treating the Kronig–Penney one-dimensional model, cf. Figure 1, in the limit of walls with vanishing thickness, but infinite height, possessing an arbitrary opacity, Ω. For Ω=∞, we have a model for an insulator; for Ω=0, we have a model for a metal. Independently of Ω, we find that the probability to find an electron pair is maximal, $P_{\max}$, Equation (2), when it is located in a domain D roughly corresponding to that between two consecutive walls. Trivially, for Ω=∞, $P_{\max}=1$, the electron pair cannot escape from the domain. For any finite Ω, there is a chance that the electrons tunnel through the walls, and thus $P_{\max}$ decreases. However, even when the walls disappear (for Ω=0), one obtains a non-negligible value for $P_{\max}$.

The calculations have revealed another feature — namely, that, as Ω→0, the domain D maximizing the probability, $D_{\max}$, Equation (3), is not unique (if we allow for some small deviation from the optimal result; this deviation vanishes at Ω=0). In the regime Ω→0, a translation of the maximal probability domain has almost no effect on the probability: the “mobility” of the electron pairs is increased in parallel with the reduction in the band gap. In this sense, we can say that the present results overlap with Pauling’s picture of the electronic structure in metals.

### 4.2. Speculation

Of course, the picture in three dimensions, 3D, is different. Furthermore, atoms have cores, and the Pauli principle produces spherical “walls” around the nuclei. Nevertheless, we can expect a similar behavior, in the sense that cores can be avoided in 3D, and “channels” exist through which displacement hardly changes the probability to find an electron pair in them. This is supported by calculations with the electron localization function [7], ELF — see, e.g., the change between the insulating α-Sn and the metallic β-Sn [8].

### 4.3. Analogy

The phenomenon of deforming the optimal domain, $D_{\max}$, without noticeable effect on the probability does not show up only in metals. For example, it is present also in atoms producing atomic shells: we can define a region for an electron pair, and any rotation produces another domain with strictly the same probability, (see, e.g., Ref. [9]). Such an effect can exist also in molecules — e.g., for an electron pair of the CC (“banana”) bond in acetylene when rotating around the molecular axis. What seems to be specific to metals is the “transport” property of the maximum probability domain, $D_{\max}$: it can “travel” through the whole crystal. 

## Data Availability

The Mathematica notebook producing the data is available on request from the author.
